# Can ID Repetitive Elements Serve as *Cis*-acting Dendritic Targeting Elements? An *In Vivo* Study

**DOI:** 10.1371/journal.pone.0000961

**Published:** 2007-09-26

**Authors:** Tasneem Khanam, Carsten A. Raabe, Martin Kiefmann, Sergej Handel, Boris V. Skryabin, Jürgen Brosius

**Affiliations:** Institute of Experimental Pathology, University of Münster, Münster, Germany; Wellcome Trust Sanger Institute, United Kingdom

## Abstract

Dendritic localization of mRNA/RNA involves interaction of *cis*-elements and *trans*-factors. Small, non-protein coding dendritic BC1 RNA is thought to regulate translation in dendritic microdomains. Following microinjections into cultured cells, BC1 RNA fused to larger mRNAs appeared to impart transport competence to these chimeras, and its 5′ ID region was proposed as the *cis*-acting dendritic targeting element. As these ID elements move around rodent genomes and, if transcribed, form a long RNA stem-loop, they might, thereby, lead to new localizations for targeted gene products. To test their targeting ability *in vivo* we created transgenic mice expressing various ID elements fused to the 3′ UTR of reporter mRNA for Enhanced Green Fluorescent Protein. *In vivo*, neither ID elements nor the BC1 RNA coding region were capable of transporting EGFP RNA to dendrites, although the 3′ UTR of α-CaMKII mRNA, an established *cis*-acting element did produce positive results. Other mRNAs containing naturally inserted ID elements are also not found in neuronal dendrites. We conclude that the 5′ ID domain from BC1 RNA is not a sufficient dendritic targeting element for mRNAs *in vivo*.

## Introduction

Dendritic localization of RNAs and localized protein synthesis in neuronal dendritic and axonal processes have received increased attention due to their relevance to synaptic plasticity. There are several hundred mRNAs known to be dendritically localized in neurons [1], including MAP 2a/b [2], the α-subunit of Ca^2+^/ calmodulin-dependent protein kinase type II (α-CaMKII) [3], the cytoskeleton-associated protein Arc [4], [5], protein kinase Mζ (PKMζ) [6], and some non-protein-coding RNAs (npcRNAs) such as, ribosomal RNAs [7], earlier seen as polyribosomes [8]–[11], BC1, BC200, and G22 RNAs [12]–[14], tRNAs [15], and even miRNAs [16]. Although many RNA species are located in dendrites, molecular mechanisms of RNA transport are not completely understood. A paucity of sequence commonality speaks against shared primary structures being responsible for this function. There are only a few reports characterizing *cis*-elements (dendritic targeting element, DTE) in dendritically localized mRNAs, such as the 3′ UTRs of α-CaMKII [17]–[19]and MAP2 mRNAs [20] and the 5′ ID domain of BC1 RNA [21]. Presumably, specific *trans*-acting factors bind *cis* elements of transported RNAs, enabling them to associate with cytoskeletal filaments and be transported with the aid of motor proteins [22], [23].

Rodent BC1 RNA is a npcRNA expressed almost exclusively in neuronal tissue, where it is transported to neuronal dendrites [12]. BC1 RNA, especially when devoid of bound proteins, inhibits translation *in vitro* and in transfected cells [24]–[26]. To study subcellular transport, Muslimov et al. [21] microinjected BC1 RNA and fragments thereof into the cytoplasm of sympathetic neurons in culture. They demonstrated that the dendritic targeting competence of BC1 RNA resides in its 5′- domain and that BC1 RNA imparts dendritic targeting competence to non-dendritic mRNAs when inserted into their 5′ or 3′ UTRs.

BC1 RNA serves as a master gene for the amplification, via retroposition, of a subclass of short interspersed repetitive elements (SINEs), termed ID1 elements, in rodents [27]. Retroposition is a genomic process that involves reverse transcription of such master RNAs [28]. The resulting cDNA copies are integrated more or less randomly into the host genome and usually are transcriptionally silent, due to the lack of upstream promoter elements [14], [29]. In the rat, one or possibly a few, RNA polymerase III-transcribed ID elements gave rise to additional ID subfamilies, ID2, ID3, and ID4, recognizable by a few diagnostic nucleotide changes [27], [30], and the rat genome now harbors an estimated 130,000 ID elements. In addition, non-autonomous transcription occurs as parts of larger RNA polymerase II-transcribed hnRNAs and mRNAs.

**Figure 1:**
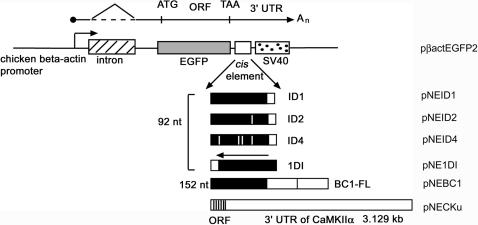
Schematic representation of transgenic transcripts and chimeric constructs containing EGFP plus various *cis* elements. The *cis* elements (ID1, ID2, ID4, 1DI) were inserted downstream (within the 3′ UTR) of the EGFP cDNA, and followed by polyadenylation signals from SV40 (spotted box). The chicken β-actin promoter was used to drive transcription of the chimeric mRNAs. The promoter harbors an intron of about 900 bp (hatched box). The various ID elements are represented by black rectangular boxes, with short A-rich tails (white boxes). ID1 corresponds to the 5′domain of BC1 RNA and the other ID elements differ from ID1 by mutations indicated by white lines within the black boxes, 1DI corresponds to ID1 in the reverse orientation. BC1-FL (full length BC1 RNA) is indicated by a black rectangular box (5′ ID domain) fused to two open boxes (representing the central A-rich region and 3′ non-repetitive region). The 3′ UTR of α-CaMKII mRNA, used as a positive control, is represented by a long rectangular open box and part of the ORF by a striped box (not drawn to scale). The labels to the right refer the respective plasmids.

Transposed elements, including SINEs, potentially impart functionality to targeted genes, for example, as promoter/enhancer elements [31], [32], polyadenylation signals [33], [34], and alternative splice sites [35], [36] that sometimes lead to exaptations at the genomic level [37]. Given our knowledge of their dendritic targeting capacity *in vitro* [21], if ID elements are retropositionally inserted into the UTRs of targeted mRNAs, they might influence their subcellular localization. To test whether mRNAs harboring the 5′ ID domain of BC1 RNA, the ID2 or ID4 elements, as defined by Kim et al. [27], [30], or full length BC1 RNA could target larger mRNAs (in this case, EGFP mRNA) to dendrites *in vivo*, we created transgenic mice expressing the corresponding transgenes in neurons (Figure 1, Supplementary Figure S1). We then used *in situ* hybridization to examine their neuronal subcellular localization. As a positive control, we expressed EGFP mRNA with the 3′ UTR of α-CaMKII mRNA containing the DTE [18] (Figure 1, Supplementary Figure S1).

## Results

### All Chimeric mRNAs are Expressed in the Brain

To analyze the expression of the chimeric mRNAs, total RNA was extracted from brains of the respective transgenic mice and from wild type mice. Northern blot analysis with EGFP probes revealed expression of chimeric EGFPs in the brains of all transgenic, but not wild type mice (Supplementary Figure S2).

### ID Elements, including the 5′-ID Domain of BC1 RNA, do not Target EGFP mRNA to Dendrites *in vivo*

**Figure 2:**
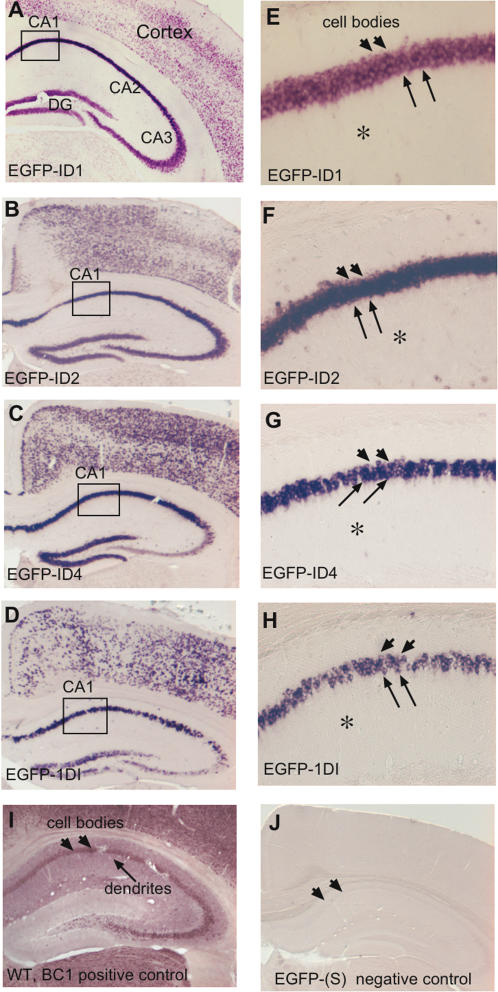
ID elements do not mark EGFP reporter mRNA for dendritic transport. *In situ* hybridization using a digoxigenin-labeled antisense (AS) EGFP probe on coronal brain sections from the various transgenic mice. *A–D,* There are intense hybridization signals in the cell bodies of neurons in the hippocampus and cortex for the ID1, ID2, ID4 and 1DI chimeric RNAs; however, there is no staining observed in the corresponding dendritic fields. *E–H*, Higher magnifications of the CA1 regions of hippocampus reveal intense staining in the cell bodies (arrowheads) but only weak staining, if at all, in only the very proximal-most regions of dendrites (arrows) and none in the distal dendritic fields (asterisks). *I,* An example of dendritic staining; brain sections from wild type mice were hybridized with a unique probe for BC1 RNA. *J*, Control hybridizations with the EGFP sense (S) probe did not produce any signals. Boxes in *A–D* represent the areas shown at higher magnification in *E–H*, respectively.

To monitor subcellular localization of chimeric RNAs, *in situ* hybridizations were performed on coronal sections through the hippocampus of transgenic mouse brains with DIG-labeled riboprobes complementary to EGFP. The various transgenes showed similar expression patterns; the chimeric RNAs, EGFP-ID1, EGFP-ID2, EGFP-ID4 and EGFP-1DI, were highly expressed in the CA1, CA2, and CA3 regions of the hippocampus, in the dentate gyrus, and various cortical layers (Figure 2A–D). To our surprise, hybridization signals for all constructs containing ID elements (in either orientation), were restricted to the cell bodies of the neurons. Only very slight signals, if at all, were observed in the very proximal-most regions of the dendrites, but clearly no hybridization signals were present within the dendritic fields of the hippocampus (Figure 2E–H). An example of dendritically localized BC1 RNA is shown in *in situ* hybridizations for BC1 RNA in a wild type mouse (Figure 2I). Hybridization with a sense probe for EGFP in transgenic mice produced no specific staining (Figure 2J).

### BC1 RNA does not Transport EGFP mRNA to Dendrites *in vivo*

**Figure 3:**
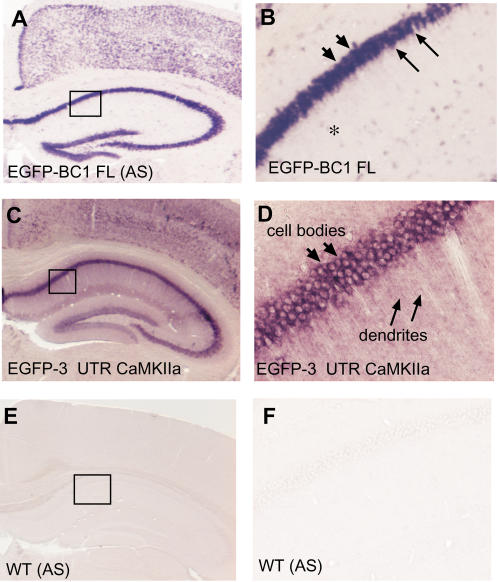
The 3′ UTR of α-CaMKII targets EGFP reporter mRNA to dendrites, whereas full-length BC1 RNA does not. *In situ* hybridization with a DIG-riboprobe against EGFP in coronal brain sections through the hippocampus of transgenic mice harboring chimeric RNAs with EGFP plus full length BC1 RNA or the 3′ UTR of α-CaMKII mRNA (positive control). *A*, EGFP-BC1 RNA was restricted to neuronal cell bodies in the hippocampus and cortex (arrowheads), and no significant labeling was observed in the dendritic regions of these cells. *B*, Higher magnification revealed dendritic labeling only in the very proximal-most region of the CA1 pyramidal cell dendrites (arrows), if at all. *C*, The EGFP- α-CaMKII 3′ UTR chimeric RNA was detected both in cell bodies and dendrites of neurons in the hippocampus and cortex. *D*, Higher magnification revealed somatic staining (arrowheads) and labeling up to the distal ends of CA1 pyramidal cell dendrites in the hippocampus (arrows). *E-F*, There were no specific hybridization signals from AS probes in wild type mice, or from sense probes in the transgenic mice (data not shown). Boxes in *A* and *C* represent the areas shown at higher magnification in *B* and *D*, respectively.

As we did not observe dendritic targeting of reporter mRNA by any of the ID elements in transgenic mice, we reasoned that, possibly, the 5′ domain alone is insufficient to impart targeting competence to the non-dendritic EGFP mRNA *in vivo*. Perhaps other domains of BC1 RNA, such as the adenosine-rich region or the non-repetitive 3′ domain, might be required as well. Hence, we examined whether full-length BC1 RNA can target EGFP mRNA to dendrites, as had been shown by microinjections of a BC1 RNA-GFP mRNA chimera or fusions of the entire BC1 RNA with *Drosophila melanogaster* bicoid mRNA in cultured primary neurons [21]. *In situ* hybridization on sections of transgenic mouse brains expressing EGFP-BC1 RNA revealed that the chimeric RNA with full length BC1 RNA was also confined to neuronal cell bodies (Figure 3A). Once more, apart from the proximal-most regions, there was clearly no signal observed in the dendritic fields of the hippocampus or in the dendrites of neurons in other areas of the brain (Figure 3A,B). Taken together, these findings show that neither the 5′ region of BC1 RNA (ID1), nor full length BC1 RNA itself can impart dendritic targeting competence to non-dendritic mRNAs *in vivo*, in contrast to data on full-length BC1 RNA from microinjection studies.

### The 3′ UTR of α-CaMKII Targets EGFP to Dendrites

As we did not detect chimeric EGFP RNAs fused to either ID elements or full length BC1 RNA in dendrites, it was necessary to demonstrate that the EGFP mRNA reporter had the *potential* to be transported to dendrites and, for example, did not contain negative elements that would suppress dendritic transport. There are several reports showing that the 3′ UTR of α-CaMKII (3.1 kb) harbors *cis*-acting element(s) sufficient for imparting extrasomatic localization to reporter mRNAs [17]–[19]. We tested therefore, whether the 3′ UTR of α-CaMKII could serve as a *cis*-acting dendritic targeting element in our system by generating transgenic mice expressing chimeric EGFP mRNA containing the 3′ UTR of α-CaMKII. *In situ* hybridization on coronal sections of the corresponding transgenic mouse brains revealed signal in cell bodies *and* throughout the full extent of the dendritic fields of hippocampal CA1, CA2, and CA3 regions as well as various layers of the cortex (Figure 3C, D. In conclusion, in the *in vivo* experimental system employed here this reporter mRNA can be transported to dendrites and can be effectively used to study the dendritic targeting competence of potential *cis*-acting elements of various dendritic mRNAs/npcRNAs *in vivo*. Neither sense nor antisense EGFP probes generated hybridization signals in wild type mice (Figure 3E,F), and the sense EGFP probe also did not hybridize to sections from transgenic mice (data not shown).

### “Natural” *in vivo* Experiments

To further confirm our unexpected data, we searched for natural insertions of ID elements into 3′ UTRs of mRNAs for which *in situ* hybridization data in brain are published. As certain structural features in the ID stem of BC1 RNA were shown to be crucial for dendritic transport *in vitro* [38], we chose mRNAs with ID elements closely resembling the 5′ stem of BC1 RNA. We examined two examples in the literature in which ID1 elements (Figure S3 and Figure S4) are inserted into the 3′ UTR of mRNAs. *In situ* hybridization of GIRK2 (Accesion # AB073753) on rat brain coronal sections revealed labeling only in the cell bodies of hippocampal CA1, CA2, and CA3 neurons [39]. Similarly, *in situ* hybridization of synArfGEF mRNA (Accesion # AB057643) on adult rat brain sections revealed labeling only in the cell bodies of CA1 pyramidal cells in the hippocampus [40]. Furthermore, *in situ* hybridization on rat hippocampal primary cultures also revealed labeling in the cell bodies and to a much lesser extent only in the very proximal dendrites [40], similar to our observations. Finally, we searched for mouse mRNAs with ID elements in their 3′ UTRs for which *in situ* hybridizations were available in databases (http://www.brain-map.org). We selected one example with an ID sequence similar to the ones we used in our transgenic mice. In mouse brain, *in situ* hybridization of Pex13 mRNA (Accession # NM_023651; see Figure S3 and Figure S4) revealed signal only in the cell bodies of neurons in the hippocampus and dentate gyrus and in the cortex. That none of these ID elements, found naturally in the UTRs of various RNAs, conveyed dendritic targeting properties to their respective mRNAs further supports our data in transgenic mice showing that ID elements alone could not serve as *cis*-acting targeting elements for the non-dendritic reporter mRNA, EGFP.

## Discussion

Neurons are extremely polarized cells with distinct morphologies, unusually long dendritic and axonal processes, structures that are crucial to their ability to communicate at long distances and to form complex cellular networks of the nervous system [41]. A number of mRNA transcripts are transported to dendrites and are translated locally in response to various specific stimuli, thus translation is locally regulated, which in turn can modulate synaptic plasticity. Small non-protein-coding RNAs, such as BC1 RNA, BC200 RNA, G22 RNA, and miRNAs are thought to play roles in local regulation of translation [12]–[14], [16], [24]–[26].

In recent years, determining the subcellular localization of RNAs has emerged as an important field [42]–[45]. Various approaches have been used to carry out these studies, including microinjections of radiolabeled or chimeric RNAs into the cell bodies of neurons [21], [46], cell transfections with plasmids harboring the templates to synthesize chimeric mRNAs of interest [18], [20], and transgenic mice expressing the given RNAs [17]. However, it is unclear if all of these approaches are reliable, as some of them are reported to have technical limitations [46]. For example, there are conflicting reports on the dendritic targeting elements of the most extensively studied candidate, α-CaMKII mRNA. In a transgenic mouse model [17] the entire 3′ UTR of the α-CaMKII mRNA (3.1 kb) was used to transport a *lacZ* reporter mRNA into dendrites. By transfecting primary hippocampal cultures with fragments of the 3′ UTR, it was further shown that the 5′-most 94 nt of the UTR contained a *cis*-acting element capable of dendritic transport, and that a shorter, 30 nt sequence (positions 26–58) within these 94 nucleotides seems sufficient to transport GFP reporter mRNA into dendrites [19]. By contrast, a third report also using transfected primary hippocampal cultures showed that 1.2 kb of the UTR at positions 1481–2708 is more efficient at dendritic localization of the EGFP reporter mRNA [18]. This exemplifies the importance of transgenic animal models, which although more labor intensive, are closest to the natural physiological conditions.

Mouse BC1 RNA is 152 nt long and has a tripartite structure [47]. The 5′- domain or ID portion of BC1 RNA has been reported to carry a code [48] for dendritic transport of BC1 RNA [21], [49]. BC1 RNA is a master gene for ID repetitive elements [27], SINEs that are randomly shotgunned around the genome via retroposition. As many retronuons have the potential to add functions to a gene [37], it was reasonable to establish a working hypothesis that ID elements might alter subcellular localizations of mRNAs from somatic alone to somatic *and* dendritic if, for example, they became integrated within the UTR of the mRNA. To assess whether insertion of an ID element has the potential to impart dendritic localization, we generated transgenic mice with constructs that enabled us to test a somatic mRNA for potential acquisition of dendritic transport competence by inserting ID elements or the entire coding region of BC1 RNA into the 3′ UTR of the reporter mRNA. However, none of the chimeric reporter constructs harboring either the 5′ stem of BC1 RNA or other classes of ID elements (Figure 2A–H) could be detected in distal or even intermediate parts of neuronal dendrites. Even the construct harboring the entire BC1 RNA coding region, including the central A-rich region and the 3′ unique region, remained in neuronal cell bodies (Figure 3A,B). This construct is very similar to the one that was observed in distal parts of dendrites following microinjection into cell bodies of cultured neurons [21]. The discrepancy cannot be readily explained, especially considering that a chimera containing the same reporter plus the 3′-UTR of α-CaMKII mRNA *was* efficiently transported to the distal dendrites of both hippocampal and cortical pyramidal cells (Figure 3C,D).

Even though the expression and subcellular localization of chimeric mRNA constructs within transgenic mice occur under much more physiological conditions, we have considered a couple of ideas that might account for the discrepancies between our results and those of others examining the targeting of BC1 RNA. Several limitations of microinjecting mRNAs into cultured oligodendrocytes were discussed [46]. It is possible that in our case the expressed transgenes overloaded the respective transport system, however, unless BC1 RNA is transported via a different process, the distal dendritic transport of EGFP mRNA with the UTR of α-CaMKII speaks against this. And, if the transport system *were* overloaded, one would still not expect sole transport of the endogenous RNAs to the complete exclusion of the transgenic RNA. In support of this, in transgenic mice expressing the primate BC200 or G22 RNAs [14], both the endogenous BC1 RNA and the transgenes were transported to the distal ends of dendrites (M. Bundman, T. Rozhdestvensky, J.B., unpublished observations).

Importantly, in transgenes the sizes of RNAs/mRNAs do not appear to play a role in dendritic targeting, as we have shown recently that the BC200 RNA (200 nt) transgene and a recently discovered dimeric Alu-derived G22 RNA (300 nt) transgene [14], as well as the EGFP-α-CaMKII 3′UTR mRNA (4.6 kb), are all effectively transported to dendrites in transgenic mice. However, it would be interesting to investigate whether the chimeric RNAs harboring ID or BC1 RNA as *cis* elements would be transported to dendrites under other physiological conditions as observed for the *cis* element in α-CaMKII [19]. Inspection of the constructs revealed one difference between this and the other constructs. Most likely, the EGFP-α-CaMKII 3′UTR mRNA uses the α-CaMKII polyadenylation signal at position 4306–4311 and not the downstream signals at positions 4553–4558 and 4582–4587 located in the cassette contributed by SV40. The remote possibility exists that the sequence between the α-CaMKII and SV40 polyadenylation signals has a negative impact on dendritic transport. Analysis of other naturally occurring mRNAs with ID elements (see below), however, renders this scenario quite unlikely.

Recently, it was shown in microinjected sympathetic neurons that certain secondary structural features of the 5′ (ID) stem-loop of BC1 RNA were important for dendritic transport [38]. Removal of a single bulged uridine residue (position 22) led to complete loss of dendritic localization, while ablation of a GA kink-turn motif prevented distal dendritic targeting. Removal of a basal internal loop or the primary sequence of the terminal loop did not affect transport. We examined mRNAs with natural insertions of ID elements for which *in situ* hybridization data are available. The three ID elements were in the correct orientation with respect to the mRNA and their stem structures closely resembled that of BC1 RNA (Figure S4). None of these mRNAs with natural ID insertions were detected in dendrites, apart from perhaps areas very close to cell bodies, as was the case with our chimeric constructs.

In conclusion, although some of the numerous retroposed SINE insertions add functionality to targeted genes, their RNA transcripts, or their protein products, we could not demonstrate under physiological conditions in transgenic mice that ID elements serve as potential *cis*-acting elements for neuronal dendritic transport, as has been shown in microinjection experiments in cultured neurons. Even though the 3′ UTR of α-CaMKII mRNA was capable of transporting the chimeric EGFP mRNA into distal portions of dendrites, none of the ID or BC1 insertions was capable of targeting the EGFP reporter mRNA to dendrites.

## Materials and Methods

### Reporter mRNA

Our experiments required a reporter mRNA that could be expressed in neurons where BC1 RNA had been shown to be dendritically localized, such as the hippocampal pyramidal cells [12]. At the same time, the unmodified reporter mRNA was required to be restricted to cell bodies. We chose as reporter, mRNA encoding enhanced green fluorescent protein (EGFP). Transcription was driven from the chicken β-actin promoter. After splicing a ∼900 nt intron from the β-actin-derived hnRNA, the 5′ UTR contained ∼80 nt of the chicken β-actin sequences as well as a polylinker region of about 50 nt. Adjacent to the ORF, the 3′ UTR begins with 20 nt of polylinker sequences and ∼196 nt contributed by a cassette containing the polyadenylation signal of SV40. We then engineered “retroposition events” into the 3′ UTR of the reporter transcript. Retroposons included full length BC1 RNA, members of the younger ID element families, ID1, ID2, and the older ID4. Since ID elements tend to integrate in both forward and reverse orientations, we also examined the insertion of an ID1 element in the reverse orientation. (1DI; Figure. 1, Figure S1).

### DNA Constructs for generation of transgenic mice

Constructs for the various ID elements were generated in pβactEGFP2 (kindly provided by Dr. Stefan Kindler, Hamburg). The vector transcribes an EGFP mRNA originally derived from pEGFP-N1 (Clontech Laboratories) driven by the chicken β-actin promoter from pβact-16 [50]. In the plasmids, pNEID1, pNE1DI (reverse orientation), pNEID2, pNEID4, pNEBC1, the ID elements ID1, ID1 in the reverse orientation, ID2, ID4, and full length BC1 RNA coding region were inserted into the *Xba*I and *Bcl*I restriction sites of a linker region (31 bp) downstream of the EGFP coding region, respectively. The inserts were generated using a PCR-based approach with appropriate restriction sites. The inserts for pNEID1 and pNEID2 were generated from pTubID1 and pTubID2 using the following primer pair: ID1/ID2for 5′-gcgtgctctagaGGGGTTGGGGATTTAGCTCAG-3′ and IDRev 5′-gcgcgtactagtCAAAACCAAGAAAAAAAGCCTCGAC; for pNEID4: ID4for 5′-gcgtgctctagaGGGGcTGGGGATTTAGCTCAG-3′ and IDRev, from pTubID4 template; for pNE1DI: BC1rev–for 5′-gcgtgctgatcaGGGGTTGGGGATTTAGCTCAG-3′ and BC1rev–rev 5′-gcgcgtagatctCAAAACCAAGAAAAAAAGCCTCGAC-3′ from pTubID1; for pNEBC1: 1D1/ID2for and BC1uni 5′-cgcgcaACTAGTTTTCCAACACACACGGTC-3′. Bases in upper case correspond to ID element or BC1 RNA sequences and those in lower case to flanking adapter sequences containing restriction sites for cloning. The plasmid pNECKu, containing part of the α-CaMKII mRNA ORF as well as the 3.1 kb of UTR [18] was obtained from Dr. S. Kindler. All relevant parts were confirmed by sequencing with the BigDye® Terminator Cycle Sequencing Ready Reaction Kit, version 2.0 (PE Applied Biosystems). Reaction products were run on an ABI Prism® 3700 capillary sequenator or an ABI Prism® 377 DNA sequenator (Perkin Elmer). Primary structures of the relevant plasmid fragments and/or deduced sequences of processed hybrid reporter mRNAs are deposited in GenBank (accession numbers EU056358- EU056364) and are shown in Supplementary Figure 1. DNA fragments for generating transgenic mice were released from the respective constructs using *Pvu*I and *Nru*I/*Eco*RI restriction endonucleases, diluted to 3 ng/µl, and injected into the pronuclei of FVB oocytes [51], which were subsequently transferred into the oviducts of 0.5 day, pseudopregnant, CD1 foster mice.

### Tail biopsy and Southern Hybridization

To determine which mice were transgenic, genomic DNA from the tails of 3-week-old mice was extracted as previously described [52], and analyzed by Southern blot hybridization with a 723 bp α-^32^P-labeled probe specific for EGFP DNA sequences as previously described [14].

### Total RNA extraction and Northern blot analysis

Transgenic and wild type mice were anaesthetized with halothane and transcardially perfused with PBS. The brains were divided into three parts by a coronal cuts through the optic chiasm and just in front of the cerebellum. The anterior and posterior portions were used for RNA isolation and the middle portion containing the hippocampus was placed in 4% paraformaldehyde to fix the tissue for *in situ* hybridization. Total RNA from brain was extracted using TRIzol reagent (Gibco BRL) according to the manufacturer's instructions. Northern hybridization with an α-^32^P-labeled probe complementary to the EGFP region was carried out as previously described [14].

### DIG-labeled in situ hybridization

To determine the subcellular localization of chimeric mRNAs, non-radioactive *in situ* hybridization on transgenic mouse brain sections was carried out as previously described [14]. Digoxigenin (DIG)-labeled riboprobes specific for EGFP were generated from plasmid pBluEGFP, which contained 723 nucleotides encoding EGFP cloned into pBluescript KS(+) between the *Kpn*I and *Sac*I restriction sites of the multiple cloning sites. The probe specific for BC1 RNA was generated as previously described [12]. The DIG-labeled sense and antisense ribo-probes were synthesized *in vitro* using T7 or T3 RNA polymerases, after linearization with *Kpn*I and *Sac*I restriction endonucleases, respectively, according to the manufacturer's instructions (Roche Molecular Biochemicals).

### ID Sequence Search

BLAST searches in NCBI Rat refseq_rna database were carried out using the sequence of the ID stem of BC1 RNA as the query sequence. Advanced BLAST options were used to limit these results to RNA entries that were expressed in brain. Presence and correct orientation of the repeats were then analyzed with the local RepeatMasker. Candidate mRNAs were then screened for available *in situ* data in the published literature.

In addition, mouse 3′UTRs of the known gene dataset were downloaded from table browser at: http://genome.cse.ucsc.edu/cgi-bin/hgTables and were analyzed for repeats utilizing the local RepeatMasker. (Smit, AFA, Hubley, R & Green, P. *RepeatMasker Open-3.0*.1996-2004 http://www.repeatmasker.org). The output was separately analyzed for BC1- and ID-derived repeats. 3′ UTRs that contained ID- or BC1-derived SINE elements in the correct orientation were checked manually for the presence of Allen Brain Atlas entries *via* the BLAT server at http://genome.cse.ucsc.edu/cgi-bin/hgBlat?command = start. All cases for which the *in situ* hybridization probe included repetitive parts were excluded from further analysis. RNA structure predictions were performed using the MFOLD software at: http://bioweb.pasteur.fr/seqanal/interfaces/mfold-simple.html

## Supporting Information

Figure S1 The nucleotide sequence of a construct used to generate transgenic mice and deduced sequences of processed hybrid reporter mRNAs. (A) The basic construct, as well as relevant parts of the reporter mRNAs (B–G). Vector sequences in (A) flanking the sequence shown are in lower case. The chicken β-actin promoter sequence and the 5′ UTR are in upper case; the TATA box and transcriptional start site (A) are underlined. The intron is shown in lower case and the polylinker regions in upper case italics. The ORF of enhanced green fluorescent protein (EGFP) is shaded in grey. The various test sequences, which are potential cis-acting element(s) (here, full length BC1 RNA) are in bold and embedded between polylinker sequences (italic). The subsequent UTR and polyadenylation signals (underlined) from SV40 virus are shown in upper case. In (B–G) the salient parts of the reporter mRNAs are shown beginning with the transcription start sites (underlined). The introns are removed and UTRs are depicted in upper case (polylinker contributions are in italic). The various inserts, full-length BC1 RNA (B), ID1 (C), ID2 (D), ID4 (E), ID1 in opposite orientation (F), and the 3′ UTR of α-CaMKII (G) are in bold type. The C-terminal portion of the α-CaMKII ORF is shaded in grey. The remaining portion of the 3′ UTR of the reporter mRNA is in regular upper case with polyadenylation signals underlined. The poly(A) tails (usually 10–20 nucleotides downstream from the polyadenylation signal are not shown. The reporter mRNA in (G) is probably terminated after the first polyadenylation signal.
(0.06 MB DOC)

Figure S2 Chimeric mRNAs are Expressed in the Brain Northern blot hybridization of total RNA extracted from brain tissue of transgenic and wild type mice. The extracted RNAs were separated on a 1.2% denaturing agarose gel and hybridized with 32P-labeled probe complementary to EGFP. Specific signals corresponding to the expected sizes was observed for chimeric RNAs. As a loading control the membrane was hybridized with a probe complimentary to α-tubulin mRNA.
(7.73 MB DOC)

Figure S3 Sequences of ID elements from our chimeric RNAs and from published examples found in the UTRs of other genes. Alignment of ID elements to the one found in the 5′ domain of dendritic BC1 RNA in rat (Rno, top line) and mouse (Mmu, second line). Nucleotides identical to the ID domain of rat BC1 RNA are shown as dots, nucleotide replacements by the corresponding changes, and deletions by hyphens. The areas thought to be vital for dendritic transport [38] are in bold lettering. The ID1, ID2, and ID4 elements all fold in the same manner as does the corresponding BC1 RNA domain [47].
(0.02 MB DOC)

Figure S4 Secondary structures of ID elements. Secondary structures of the ID domains found in other genes and for which brain in situ hybridization data are available in the literature or in databases, respectively. Nucleotides corresponding to those thought to be vital for dendritic transport (see above) are highlighted in red and deviations from the ID element in BC1 RNA in blue. The corresponding nucleotides in BC1 RNA are indicated by arrows.
(9.35 MB TIF)
