# Supplementary material for: Can ID Repetitive Elements Serve as Cis-acting Dendritic Targeting Elements? An In Vivo Study
Source: PLoS One. 2007 Sep 26;2(9):e961. doi: 10.1371/journal.pone.0000961 (PMC1978531; doi:10.1371/journal.pone.0000961)
Supplement: Figure S1 — The nucleotide sequence of a construct used to generate transgenic mice and deduced sequences of processed hybrid reporter mRNAs. (A) The basic construct, as well as relevant parts of the reporter mRNAs (B–G). Vector sequences in (A) flanking the sequence shown are in lower case. The chicken β-actin promoter sequence and the 5′ UTR are in upper case; the TATA box and transcriptional start site (A) are underlined. The intron is shown in lower case and the polylinker regions in upper case italics. The ORF of enhanced green fluorescent protein (EGFP) is shaded in grey. The various test sequences, which are potential cis-acting element(s) (here, full length BC1 RNA) are in bold and embedded between polylinker sequences (italic). The subsequent UTR and polyadenylation signals (underlined) from SV40 virus are shown in upper case. In (B–G) the salient parts of the reporter mRNAs are shown beginning with the transcription start sites (underlined). The introns are removed and UTRs are depicted in upper case (polylinker contributions are in italic). The various inserts, full-length BC1 RNA (B), ID1 (C), ID2 (D), ID4 (E), ID1 in opposite orientation (F), and the 3′ UTR of α-CaMKII (G) are in bold type. The C-terminal portion of the α-CaMKII ORF is shaded in grey. The remaining portion of the 3′ UTR of the reporter mRNA is in regular upper case with polyadenylation signals underlined. The poly(A) tails (usually 10–20 nucleotides downstream from the polyadenylation signal are not shown. The reporter mRNA in (G) is probably terminated after the first polyadenylation signal. (0.06 MB DOC) [file pone.0000961.s001.doc]

# A

EGFP-BC1FL DNA construct (pNEBC1)

tgggaagggcgatcggtgcgggcctcttcgctattacgccagctggcgaaagggggatgtgctgcaaggcgattAAGTTGGGTAACGCCAGGGTTTTCCCAGTCACGACGTTGTAAAACGACGGCCAGTGCCAAGTTGGGATCTTTGCATTGGCCCACGGCTCTCAGGATGGGGATGCTCCCCTTCAGCACCCGGTTCCCCTTGGAAACTGATGGTCCTGGCTCTGTGGCATGGCAGTGGCACTGTGAGGAGCCCCCTACCAGCAGCACACAGTGGGGTTGGCACTGCCACGCTCCGGATGCCGCGCTCTGATCCAACCCCATAATCAAGGGAACCCGAATTGCCCCATCATTGCCCCCACCACCCCCATCCTGCCGGGCCCTCACACCCCACGCTGCCTTCTGGTGACATTCCCCAGCCCAAACCCACGGCTTCATGGCTACCGCGGGGCATTTCCCATTGCCGCCCCATTATCAGCTCTGCACACCTCCCGCTGTACCCATGCCTCGTGGCTGCCCTTCTTTGACGTATAATCTTCTAATTAATACCCGGCCTTGTCAAAGTGGAGCACAAACGTTAATTAATTCCCCAGCAGGCAGGTAATTAACAGTGTGACTCCCTTTTTGCTGCGAGTGGGGCTGATACAGAGAGATGTGGCACTATGGAGCCCACGGGGTCCTGGCACTGGGTGCCCACGGAGGTCCCCATGTGCTGCAGTGTCACCGCCTCCGAGGAGACAGTATTGTCCCTGCGGTGTCCCTGCAGCTCAGCTCTGTCCACAGGGCCACCTCCAGTTTGGAGGGGACACAATGCAGCCCCGATGCAACCCATCCTCGCAGCATCCCAGGGACAAAGACCCCACTGCAAGACCGCACACAGGGCTGGGTCCCGCTCCCCTAATATCTACAGTGCTTCTTGCATGGCCCCTTAATCAATGCAGTTAATCAGCATGCGCTCATGCACCGCTCTGGAGCTGCAAAGCCCCTCGCAGCGCTGCTCACCAACACCGCGCACCGCCCCTGCCCAGCCTGCAGCACGCGCTGCAAACAGGAAAGAAACAAAATATTGCCCAAATGTAGGCAAAGGCATTCGGCTGCCTTGACCTCCGCCGGGCCGGGCCCTGCCTGACTCAGCTCCTTACTCAGCGCTCGCTTCCTCCCTCCGGCTGCCACCGCCGCAGCGCACACCCTGACAAAGAGTGGCCCTTAACGGGCTCTGAGGTGCACCCAGCAGTGCACTCAGCAGTCCAAGGGCCGGCCTGGAGGTTTGCACCGCTACGTGCTGACATTAGCATTGAACTTGGCCCTGGGTAGTGCTGCAGGCCGGGCGGGGTGGGTGTAGAGAGTGCAGCGCGCGTTGCACCCGGTGCCCCTTCCCCTCCCTTGCATCCCAGCAGGCTGCACCCCAGCACCAGGCCCGTGCATGCATGCTCCTGGTGTTATTGCAGCCTGGTGCATGCATGCGTCTTGATGGTGCAGCGCTGTGCATGCATCCTCCTTGGTGTGTAGCAGTCTAGTGCATGCATACCCCTCGGTGTTATTGCTGCTCTGTGCACGCACGCTCATTGTATCACTTCATCCCAGTGCATGCACTCACACTGGAGCGATTGCTGCTCGGTGCACGCACACTCATTGTATCACTGCAGCTCAGTGGCTGCACGCACACCGGTGTTATTGCTGCTCGGTGCGTGCATGCACATCAGTGTCGCTGCAGCTCAGTGCATGCACGCTCATTGCCCATCGCTATCCCTGCCTCTCCTGCTGGCGCTCCCCGGGAGGTGACTTCAAGGGGACCGCAGGACCACCTCGGGGGTGGGGGGAGGGCTGCACACGCGGACCCCGCTCCCCCTCCCCAACAAAGCACTGTGGAATCAAAAAGGGGGGAGGGGGGATGGAGGGGCGCGTCACACCCCCGCCCCACACCCTCACCTCGAGGTGAGCCCCACGTTCTGCTTCACTCTCCCCATCTCCCCCCCCTCCCCACCCCCAATTTTGTATTTATTTATTTTTTAATTATTTTGTGCAGCGATGGGGGCGGGGGGGGGGGGGGCGCGCGCCAGGCGGGGCGGGGCGGGGCGAGGGGCGGGGCGGGGCGAGGCGGAGAGGTGCGGCGGCAGCCAATCAGAGCGGCGCGCTCCGAAAGTTTCCTTTTATGGCGAGGCGGCGGCGGCGGCGGCCCTATAAAAAGCGAAGCGCGCGGCGGGCGGGAGTCGCTGCGTTGCCTTCGCCCCGTGCCCCGCTCCGCGCCGCCTCGCGCCGCCCGCCCCGGCTCTGACTGACCGCGTTACTCCCACAGgtgagcgggcgggacggcccttctcctccgggctgtaattagcgcttggtttaatgacggctcgtttcttttctgtggctgcgtgaaagccttaaagggctccgggagggccctttgtgcgggggggagcggctcggggggtgcgtgcgtgtgtgtgtgcgtggggagcgccgcgtgcggcccgcgctgcccggcggctgtgagcgctgcgggcgcggcgcggggctttgtgcgctccgcgtgtgcgcgaggggagcgcggccgggggcggtgccccgcggtgcgggggggctgcgaggggaacaaaggctgcgtgcggggtgtgtgcgtgggggggtgagcagggggtgtgggcgcggcggtcgggctgtaacccccccctgcacccccctccccgagttgctgagcacggcccggcttcgggtgcggggctccgtgcggggcgtggcgcggggctcgccgtgccgggcggggggtggcggcaggtgggggtgccgggcggggcggggccgcctcgggccggggagggctcgggggaggggcgcggcggccccggagcgccggcggctgtcgaggcgcggcgagccgcagccattgccttttatggtaatcgtgcgagagggcgcagggacttcctttgtcccaaatctggcggagccgaaatctgggaggcgccgccgcaccccctctagcgggcgcgggcgaagcggtgcggcgccggcaggaaggaaatgggcggggagggccttcgtgcgtcgccgcgccgccgtccccttctccatctccagcctcggggctgccgcagggggacggctgccttcgggggggacggggcagggcggggttcggcttctggcgtgtgaccggcggggtttatatcttcccttctctgttcctccgcagCC*CCCAAGCTTGGGCCCCCGCCAGCCATGATCGATGATATCACC*ATGGTGAGCAAGGGCGAGGAGCTGTTCACCGGGGTGGTGCCCATCCTGGTCGAGCTGGACGGCGACGTAAACGGCCACAAGTTCAGCGTGTCCGGCGAGGGCGAGGGCGATGCCACCTACGGCAAGCTGACCCTGAAGTTCATCTGCACCACCGGCAAGCTGCCCGTGCCCTGGCCCACCCTCGTGACCACCCTGACCTACGGCGTGCAGTGCTTCAGCCGCTACCCCGACCACATGAAGCAGCACGACTTCTTCAAGTCCGCCATGCCCGAAGGCTACGTCCAGGAGCGCACCATCTTCTTCAAGGACGACGGCAACTACAAGACCCGCGCCGAGGTGAAGTTCGAGGGCGACACCCTGGTGAACCGCATCGAGCTGAAGGGCATCGACTTCAAGGAGGACGGCAACATCCTGGGGCACAAGCTGGAGTACAACTACAACAGCCACAACGTCTATATCATGGCCGACAAGCAGAAGAACGGCATCAAGGTGAACTTCAAGATCCGCCACAACATCGAGGACGGCAGCGTGCAGCTCGCCGACCACTACCAGCAGAACACCCCCATCGGCGACGGCCCCGTGCTGCTGCCCGACAACCACTACCTGAGCACCCAGTCCGCCCTGAGCAAAGACCCCAACGAGAAGCGCGATCACATGGTCCTGCTGGAGTTCGTGACCGCCGCCGGGATCACTCTCGGCATGGACGAGCTGTACAAGTAA*AGCGGCCGCGACTCTAGA***GGGGTTGGGGATTTAGCTCAGTGGTAGAGCGCTTGCCTAGCAAGCGCAAGGCCCTGGGTTCGGTCCTCAGCTCCGAAAAAAAAAAAAAAAAAAAAAAGACAAAATAACAAAAAGACCAAAAAAAAACAAGGTAACTGGCACACACAACCTTT***TGATCA*TAATCAGCCATATCACATCTGTAGAGGTTTTACTTGCTTTAAAAAACCTCCCACACCTCCCCCTGAACCTGAAACATAAAATGAATGCAATTGTTGTTGTTAACTTGTTTATTGCAGCTTATAATGGTTACAAATAAAGCAATAGCATCACAAATTTCACAAATAAAGCATTTTTTTCACTGCATTCTAGTTGTGGTTTGTCCAAACTCATCAATGTATCTTATCATGTCTGgatccccgggtaccgagctgtcgcgacagctcgaattcgtaatcatggtcatagctgtttcctgtgtgaaattgttatccgctcacaattccacacaacatacgagccggaagcat

B

EGFP-BC1FL processed transcript (from construct pNEBC1)

AGUCGCUGCGUUGCCUUCGCCCCGUGCCCCGCUCCGCGCCGCCUCGCGCCGCCCGCCCCGGCUCUGACUGACCGCGUUACUCCCACAGCC*CCCAAGCUUGGGCCCCCGCCAGCCAUGAUCGAUGAUAUCACC*AUGGUGAGCAAGGGCGAGGAGCUGUUCACCGGGGUGGUGCCCAUCCUGGUCGAGCUGGACGGCGACGUAAACGGCCACAAGUUCAGCGUGUCCGGCGAGGGCGAGGGCGAUGCCACCUACGGCAAGCUGACCCUGAAGUUCAUCUGCACCACCGGCAAGCUGCCCGUGCCCUGGCCCACCCUCGUGACCACCCUGACCUACGGCGUGCAGUGCUUCAGCCGCUACCCCGACCACAUGAAGCAGCACGACUUCUUCAAGUCCGCCAUGCCCGAAGGCUACGUCCAGGAGCGCACCAUCUUCUUCAAGGACGACGGCAACUACAAGACCCGCGCCGAGGUGAAGUUCGAGGGCGACACCCUGGUGAACCGCAUCGAGCUGAAGGGCAUCGACUUCAAGGAGGACGGCAACAUCCUGGGGCACAAGCUGGAGUACAACUACAACAGCCACAACGUCUAUAUCAUGGCCGACAAGCAGAAGAACGGCAUCAAGGUGAACUUCAAGAUCCGCCACAACAUCGAGGACGGCAGCGUGCAGCUCGCCGACCACUACCAGCAGAACACCCCCAUCGGCGACGGCCCCGUGCUGCUGCCCGACAACCACUACCUGAGCACCCAGUCCGCCCUGAGCAAAGACCCCAACGAGAAGCGCGAUCACAUGGUCCUGCUGGAGUUCGUGACCGCCGCCGGGAUCACUCUCGGCAUGGACGAGCUGUACAAGUAA*AGCGGCCGCGACUCUAGA***GGGGUUGGGGAUUUAGCUCAGUGGUAGAGCGCUUGCCUAGCAAGCGCAAGGCCCUGGGUUCGGUCCUCAGCUCCGAAAAAAAAAAAAAAAAAAAAAAGACAAAAUAACAAAAAGACCAAAAAAAAACAAGGUAACUGGCACACACAACCUUU***UGAUCA*UAAUCAGCCAUAUCACAUCUGUAGAGGUUUUACUUGCUUUAAAAAACCUCCCACACCUCCCCCUGAACCUGAAACAUAAAAUGAAUGCAAUUGUUGUUGUUAACUUGUUUAUUGCAGCUUAUAAUGGUUACAAAUAAAGCAAUAGCAUCACAAAUUUCACAAAUAAAGCAUUUUUUUCACUGC

C

EGFP-ID1 processed transcript (from construct pNEID1)

AGUCGCUGCGUUGCCUUCGCCCCGUGCCCCGCUCCGCGCCGCCUCGCGCCGCCCGCCCCGGCUCUGACUGACCGCGUUACUCCCACAGCC*CCCAAGCUUGGGCCCCCGCCAGCCAUGAUCGAUGAUAUCACC*AUGGUGAGCAAGGGCGAGGAGCUGUUCACCGGGGUGGUGCCCAUCCUGGUCGAGCUGGACGGCGACGUAAACGGCCACAAGUUCAGCGUGUCCGGCGAGGGCGAGGGCGAUGCCACCUACGGCAAGCUGACCCUGAAGUUCAUCUGCACCACCGGCAAGCUGCCCGUGCCCUGGCCCACCCUCGUGACCACCCUGACCUACGGCGUGCAGUGCUUCAGCCGCUACCCCGACCACAUGAAGCAGCACGACUUCUUCAAGUCCGCCAUGCCCGAAGGCUACGUCCAGGAGCGCACCAUCUUCUUCAAGGACGACGGCAACUACAAGACCCGCGCCGAGGUGAAGUUCGAGGGCGACACCCUGGUGAACCGCAUCGAGCUGAAGGGCAUCGACUUCAAGGAGGACGGCAACAUCCUGGGGCACAAGCUGGAGUACAACUACAACAGCCACAACGUCUAUAUCAUGGCCGACAAGCAGAAGAACGGCAUCAAGGUGAACUUCAAGAUCCGCCACAACAUCGAGGACGGCAGCGUGCAGCUCGCCGACCACUACCAGCAGAACACCCCCAUCGGCGACGGCCCCGUGCUGCUGCCCGACAACCACUACCUGAGCACCCAGUCCGCCCUGAGCAAAGACCCCAACGAGAAGCGCGAUCACAUGGUCCUGCUGGAGUUCGUGACCGCCGCCGGGAUCACUCUCGGCAUGGACGAGCUGUACAAGUAA*AGCGGCCGCGACUCUAGA***GGGGUUGGGGAUUUAGCUCAGUGGUAGAGCGCUUGCCUAGCAAGCGCAAGGCCCUGGGUUCGGUCCUCAGCUCCGAAAAAAAGAACCAAAAC***UGAUCA*UAAUCAGCCAUAUCACAUCUGUAGAGGUUUUACUUGCUUUAAAAAACCUCCCACACCUCCCCCUGAACCUGAAACAUAAAAUGAAUGCAAUUGUUGUUGUUAACUUGUUUAUUGCAGCUUAUAAUGGUUACAAAUAAAGCAAUAGCAUCACAAAUUUCACAAAUAAAGCAUUUUUUUCACUGC

D

EGFP-ID2 processed transcript (from construct pNEID2)

AGUCGCUGCGUUGCCUUCGCCCCGUGCCCCGCUCCGCGCCGCCUCGCGCCGCCCGCCCCGGCUCUGACUGACCGCGUUACUCCCACAGCC*CCCAAGCUUGGGCCCCCGCCAGCCAUGAUCGAUGAUAUCACC*AUGGUGAGCAAGGGCGAGGAGCUGUUCACCGGGGUGGUGCCCAUCCUGGUCGAGCUGGACGGCGACGUAAACGGCCACAAGUUCAGCGUGUCCGGCGAGGGCGAGGGCGAUGCCACCUACGGCAAGCUGACCCUGAAGUUCAUCUGCACCACCGGCAAGCUGCCCGUGCCCUGGCCCACCCUCGUGACCACCCUGACCUACGGCGUGCAGUGCUUCAGCCGCUACCCCGACCACAUGAAGCAGCACGACUUCUUCAAGUCCGCCAUGCCCGAAGGCUACGUCCAGGAGCGCACCAUCUUCUUCAAGGACGACGGCAACUACAAGACCCGCGCCGAGGUGAAGUUCGAGGGCGACACCCUGGUGAACCGCAUCGAGCUGAAGGGCAUCGACUUCAAGGAGGACGGCAACAUCCUGGGGCACAAGCUGGAGUACAACUACAACAGCCACAACGUCUAUAUCAUGGCCGACAAGCAGAAGAACGGCAUCAAGGUGAACUUCAAGAUCCGCCACAACAUCGAGGACGGCAGCGUGCAGCUCGCCGACCACUACCAGCAGAACACCCCCAUCGGCGACGGCCCCGUGCUGCUGCCCGACAACCACUACCUGAGCACCCAGUCCGCCCUGAGCAAAGACCCCAACGAGAAGCGCGAUCACAUGGUCCUGCUGGAGUUCGUGACCGCCGCCGGGAUCACUCUCGGCAUGGACGAGCUGUACAAGUAA*AGCGGCCGCGACUCUAGA***GGGGUUGGGGAUUUAGCUCAGUGGUAGAGCGCUUGCCUAGCAAGCGCAAGGCCCUGGGUUCGGUCCCCAGCUCCGAAAAAAAGAACCAAAAC***UGAUCA*UAAUCAGCCAUAUCACAUCUGUAGAGGUUUUACUUGCUUUAAAAAACCUCCCACACCUCCCCCUGAACCUGAAACAUAAAAUGAAUGCAAUUGUUGUUGUUAACUUGUUUAUUGCAGCUUAUAAUGGUUACAAAUAAAGCAAUAGCAUCACAAAUUUCACAAAUAAAGCAUUUUUUUCACUGC

E

EGFP-ID4 processed transcript (from construct pNEID4)

AGUCGCUGCGUUGCCUUCGCCCCGUGCCCCGCUCCGCGCCGCCUCGCGCCGCCCGCCCCGGCUCUGACUGACCGCGUUACUCCCACAGCC*CCCAAGCUUGGGCCCCCGCCAGCCAUGAUCGAUGAUAUCACC*AUGGUGAGCAAGGGCGAGGAGCUGUUCACCGGGGUGGUGCCCAUCCUGGUCGAGCUGGACGGCGACGUAAACGGCCACAAGUUCAGCGUGUCCGGCGAGGGCGAGGGCGAUGCCACCUACGGCAAGCUGACCCUGAAGUUCAUCUGCACCACCGGCAAGCUGCCCGUGCCCUGGCCCACCCUCGUGACCACCCUGACCUACGGCGUGCAGUGCUUCAGCCGCUACCCCGACCACAUGAAGCAGCACGACUUCUUCAAGUCCGCCAUGCCCGAAGGCUACGUCCAGGAGCGCACCAUCUUCUUCAAGGACGACGGCAACUACAAGACCCGCGCCGAGGUGAAGUUCGAGGGCGACACCCUGGUGAACCGCAUCGAGCUGAAGGGCAUCGACUUCAAGGAGGACGGCAACAUCCUGGGGCACAAGCUGGAGUACAACUACAACAGCCACAACGUCUAUAUCAUGGCCGACAAGCAGAAGAACGGCAUCAAGGUGAACUUCAAGAUCCGCCACAACAUCGAGGACGGCAGCGUGCAGCUCGCCGACCACUACCAGCAGAACACCCCCAUCGGCGACGGCCCCGUGCUGCUGCCCGACAACCACUACCUGAGCACCCAGUCCGCCCUGAGCAAAGACCCCAACGAGAAGCGCGAUCACAUGGUCCUGCUGGAGUUCGUGACCGCCGCCGGGAUCACUCUCGGCAUGGACGAGCUGUACAAGUAA*AGCGGCCGCGACUCUAGA***GGGGCUGGGGAUUUAGCUCAGUGGUAGAGCGCUUACCUAGGAAGCGCAAGGCCCUGGGUUCGGUCCCCAGCUCCGAAAAAAAGAACCAAAAC***UGAUCA*UAAUCAGCCAUAUCACAUCUGUAGAGGUUUUACUUGCUUUAAAAAACCUCCCACACCUCCCCCUGAACCUGAAACAUAAAAUGAAUGCAAUUGUUGUUGUUAACUUGUUUAUUGCAGCUUAUAAUGGUUACAAAUAAAGCAAUAGCAUCACAAAUUUCACAAAUAAAGCAUUUUUUUCACUGC

F

EGFP-1DI processed transcript (from construct pNE1DI)

AGUCGCUGCGUUGCCUUCGCCCCGUGCCCCGCUCCGCGCCGCCUCGCGCCGCCCGCCCCGGCUCUGACUGACCGCGUUACUCCCACAGCC*CCCAAGCUUGGGCCCCCGCCAGCCAUGAUCGAUGAUAUCACC*AUGGUGAGCAAGGGCGAGGAGCUGUUCACCGGGGUGGUGCCCAUCCUGGUCGAGCUGGACGGCGACGUAAACGGCCACAAGUUCAGCGUGUCCGGCGAGGGCGAGGGCGAUGCCACCUACGGCAAGCUGACCCUGAAGUUCAUCUGCACCACCGGCAAGCUGCCCGUGCCCUGGCCCACCCUCGUGACCACCCUGACCUACGGCGUGCAGUGCUUCAGCCGCUACCCCGACCACAUGAAGCAGCACGACUUCUUCAAGUCCGCCAUGCCCGAAGGCUACGUCCAGGAGCGCACCAUCUUCUUCAAGGACGACGGCAACUACAAGACCCGCGCCGAGGUGAAGUUCGAGGGCGACACCCUGGUGAACCGCAUCGAGCUGAAGGGCAUCGACUUCAAGGAGGACGGCAACAUCCUGGGGCACAAGCUGGAGUACAACUACAACAGCCACAACGUCUAUAUCAUGGCCGACAAGCAGAAGAACGGCAUCAAGGUGAACUUCAAGAUCCGCCACAACAUCGAGGACGGCAGCGUGCAGCUCGCCGACCACUACCAGCAGAACACCCCCAUCGGCGACGGCCCCGUGCUGCUGCCCGACAACCACUACCUGAGCACCCAGUCCGCCCUGAGCAAAGACCCCAACGAGAAGCGCGAUCACAUGGUCCUGCUGGAGUUCGUGACCGCCGCCGGGAUCACUCUCGGCAUGGACGAGCUGUACAAGUAA*AGCGGCCGCGACUCUAGA***GUUUUGGUUCUUUUUUUCGGAGCUGAGGACCGAACCCAGGGCCUUGCGCUUGCUAGGCAAGCGCUCUACCACUGAGCUAAAUCCCCAACCCC***UGAUCA*UAAUCAGCCAUAUCACAUCUGUAGAGGUUUUACUUGCUUUAAAAAACCUCCCACACCUCCCCCUGAACCUGAAACAUAAAAUGAAUGCAAUUGUUGUUGUUAACUUGUUUAUUGCAGCUUAUAAUGGUUACAAAUAAAGCAAUAGCAUCACAAAUUUCACAAAUAAAGCAUUUUUUUCACUGC

## G

EGFP-3’UTR of α-CaMKII processed transcript (from construct pNECKu)

AGUCGCUGCGUUGCCUUCGCCCCGUGCCCCGCUCCGCGCCGCCUCGCGCCGCCCGCCCCGGCUCUGACUGACCGCGUUACUCCCACAGCC

*CCCAAGCUUGGGCCCCCGCCAGCCAUGAUCGAUGAUAUCACC*AUGGGGCCCAAGAAGAAACGCAAAGUGGGAAGCAUGGUGAGCAAGGGCGAGGAGCUGUUCACCGGGGUGGUGCCCAUCCUGGUCGAGCUGGACGGCGACGUAAACGGCCACAAGUUCAGCGUGUCCGGCGAGGGCGAGGGCGAUGCCACCUACGGCAAGCUGACCCUGAAGUUCAUCUGCACCACCGGCAAGCUGCCCGUGCCCUGGCCCACCCUCGUGACCACCCUGACCUACGGCGUGCAGUGCUUCAGCCGCUACCCCGACCACAUGAAGCAGCACGACUUCUUCAAGUCCGCCAUGCCCGAAGGCUACGUCCAGGAGCGCACCAUCUUCUUCAAGGACGACGGCAACUACAAGACCCGCGCCGAGGUGAAGUUCGAGGGCGACACCCUGGUGAACCGCAUCGAGCUGAAGGGCAUCGACUUCAAGGAGGACGGCAACAUCCUGGGGCACAAGCUGGAGUACAACUACAACAGCCACAACGUCUAUAUCAUGGCCGACAAGCAGAAGAACGGCAUCAAGGUGAACUUCAAGAUCCGCCACAACAUCGAGGACGGCAGCGUGCAGCUCGCCGACCACUACCAGCAGAACACCCCCAUCGGCGACGGCCCCGUGCUGCUGCCCGACAACCACUACCUGAGCACCCAGUCCGCCCUGAGCAAAGACCCCAACGAGAAGCGCGAUCACAUGGUCCUGCUGGAGUUCGUGACCGCCGCCGGGAUCACUCUCGGCAUGGACGAGCUGUACAAGUAA*AGCGGCCGCUCGACGGUAUCGAUAAGCUUGAUAUCG***AAUUCGGCACGAGCUGGACUUUCAUCGAUUCUAUUUUGAAAACCUGUGGUCCCGGAACAGCAAGCCCGUGCACACCACCAUCCUGAACCCUCACAUCCACCUGAUGGGUGACGAGUCAGCCUGCAUCGCCUACAUCCGCAUCACUCAGUACCUGGAUGCGGGUGGCAUCCCCCGCACGGCCCAGUCAGAGGAGACCCGUGUCUGGCACCGCAGGGAUGGAAAAUGGCAGAUCGUCCACUUCCACAGAUCUGGGGCGCCCUCCGUCCUGCCCCAUUGAAGGACCAGGCCAGGGUCCCUGCGCUCUUGCUUCGCAGAGAUCCAUUCUUUGUCCAUGGAAUGUGGCUGCUGGCUCUCCCUUGGAUGUUGCUGGAAUUCUCCCUGUCCUAUCACCCUACCGUUGCCAUCUCUGUAUUUGCAUCAUGGAAGCCUGCUUGUUCACAGAAGCCCUCAUGACGUCACAGCGAAUGGCCAGCUCUCUCCAGCUCCACUGCCCACGCUCUUCCUGCCAGUGGGGACCUUCUUCAGGCUUGAGUACCCAGGGUGCUGGCCCCAGGAACCCCCAUCCCCUACCCACUCUUGUUAGCCUGGCCUAGCUUUGGCCCUAAAUGAGGAGGCCCCAGCUGUGCAUUUGGCAGGAAGUAAGGCAAGGAGAGGAGGGCGAGCUGUGACCAGGGCCCCUCUUGUCAGUUCCUUCCCUUCUGGGGUCCCUGGGGAAGCGUAGAUGAGCCCCUCAGUCUCCAAGCCAACUCCUUAUAAGGGAGACUGAGAGAGGAGUCAACACCAGGAACUGCUAAUCUCAUCUGCUCCCCUCUGGGCUGGCCUUGCGUUUGAUCAGACCAUCCACCAUGGGGGAAGAGAGGGAGUGGGCUCUACGGUCUGUCCAGGUACCCCACUCACUGCCUCAGUCCGUCUGUGAAGUUUGUCUCCAGUGUUGACCCACCCACCCUGCCCUUCAACGUCCUUGGAGAAUUCCAGCCUCAUCUGUCUAAGAGGAGAUCUGAUGGUGUUUCAGGGGCAAUAGCAAGCAACACUUAGGUAUCACUUCUACUUGGACGCAUGCCUUUUUACAGCCAAACCUCUGUGUAUUUCGUAAAUGGAUUUUGCGUUAACGGACAUUUAUGUGAUAACUAGACCUCUCAAGUUAUUGUAAAGAGUCGGAUGGGUUGGGAAGUGGGUGGGAAGAGGAGUGAGGGGAAGUUUUUACCCUGUUCCAGAGGGUUUUUUUUGGGGGGGAGAGUCUUUUCUGGGGUGGCCUCAGCUCCCCCACCCCCCCACCAGGGAGCACGUCUUCCUUCCAUUCUGGUCCCCAAGGUCAGCUGACAAGAUCUCUUCCAGAGCCAGCAUGACUAACACACAGUGAGUCAGGGCAGGGGAGGCCAUCAGGAUGGGAACAUCCAGACUUGGUAUCUCUGGAACUGGAGAUCUCACUACCCUACUUCCUGGGAAAUCUUUCUCCAAAUCUUCGUGCUCUCCCUCCCCAUCCCGGUGCCGCUCACUACCAUCUGGGUACCUCAUUCCCAUUGCCUCUCUCAACUCGGCCAAGCAAGAGUACCAGAAACUUAUUUUCCUACUUCACGCUGAAUUUGGGCUGGUGUGCGCUCUUAAUUGCGCUUGCCUGAUUGCCCGGCCUCCCUCCCCCAGCCCCCCCUACCCCCUUCCUCUCUUUCCCCUCUCCCUUCCUUUCUCUUUCUCUUCCUCUCUCUCCAUCUUCCUGGGUUUGGUUCUUAUAUGGAAUGCUGUGUCUCAUCCACGGGGAUCCCUUGUCUGCACUGUUUUCUUUGCAUGACUUUAUAUGCAGUAAGUAUGUUGAGAAAAAAAAGAGCAAAGACAAAAAAGAAGAAAAACACUCAGCAAAAUCAAACGACACGUUUUGGACAAAAAAUAUAAUAAUAACAUUCAAGGUUAUAUUCUCAGUGUCCAACUUGGAAUUACGUUGCUGCCUCUCUGUGCUUUUGGUCUCUGUGUGGCUGUGUUUUGCCAGCAUGAGACCCUGUCCCCUCUGGAGGUUUCUAGGGGAGGAAGAGCCGUGUGUCGGGGGGGGGGGUUGGAGACAGCUUUGUCCUCUCAGCUUUUUGGGGGGGUUGAUUGGAGCAGAAGUGGAAGGGGAUGUUUAAUCCAGAACUUUCUGGUAUUUCCCUUUCUCCCACGCAGUGAGCUAUACGCUGGGCUCUUCUCUCAAAUCCUGCUGCCCAGGGACAAGUAUAGGGUAGAAGGGUGGCCCUAUUGUCUAAGCCACUCCACUGUAGCCCUCUGCCUUUGGUAGAGACACUGCUACCCAGACCCAAGAAUGGGCCCUUGUCCCACCCCAGAUCUAGGCUUCUUCAUAAGGCUCAGCAAACUCAUUGUCCCCAGCCAUUCCCCCACUAAAGGUAAAAGAAGGUGUGGCCUUUACCAGGGGACACUGCGAUUAUCAAUCAAGCCCUCUUCAAGCCUCAGUUUCACCACCAAUGUUCCUACCCAGACUGAUGGAAGGUCAAACUAAAUGAUGUCACAAGUGCACACCAUCUUUGAGAACUUGCUGGGUUUGUCACUGGCUGGCCUUCUUAUGCACCAGGCCCGGCCAAUUCCCAUCUUUUCCCCUGUGUGCCCCCUCAUUUUCCUAUUUGGUGCCAGUCUGUUGAAGACCAGCAACAAAUGCAGGGGAAAGAAGUGUCUGGGGGCUUUGGUAGGCUUUGACCCCCCGUUCUGAUCAGAAGGGCUGUGUGGCUUUGGGUGAGUCCUGUGCCCUCCUGGGGCCUUAGUUUCCUCAGCCAGAAGAUGCCUAUGCCCUGCCUUCUGUUGGCUAACAUGCCCCUGUCCACUGUGUGCCUGUCCACAUGUGGAGAAGUGGAGGCAGGUCCCUGCCCCAGUCUGAGACGGCCCGCUCUGCAGAGGCCGCUCCUGUGGGUGGGCAGCCAACUCAUGUAGACCUUGGGACACUACAAUGGCCCCAAGGUAGCAGGCAGGGGAACUGGCAGAAAAACUGCCCUCCUCAGACAAGCUUCAUGCUCCUUCCAAAUACCCUCCAGAUGAAAAAAGAGAGAGAGAGAAACAGAGUCAAGUCACAUUUAUAGGAAAAGCAUCUCCAGGCAUAUGUGCCGUAGCUCAAAUCCUGCCCCAUGGCUUCCCCACCCCCUUCAAAGGGAGAGUCUUGGGGGAAUUUGUUUGCCAGGCCCCGUGCUGACUUCUUUGUUACUACUUGUUUAGGGUUUUGUUCUAGUUCUCUCUCUCUCUCUCUCUUUCUCUCUCUCUCUCUCUCUCUCUCUCUCUCUCUCUCUCUCUCUCUCUUUCUUUUUUAUUAUGUGGCUGUGAACUUGAAUGACCAUUGCUCAAACUUUCUGCUACUGGGGGUGGGGUGGGGGAGGGGAGAAGAGACGUCUGUUUUAUUCUUGGUGUUUUCAGUGGAAUAAAUAGCUACAAAUUUAAAAAAAAAAA***UCGGCACGAGCUCGUGCCGAAUUCCUGCAGCCCCGGGGGAUCCACUAGUUCUAGAGCGGCCGCGACUCUAGAUAAGUAAUGAUCA*UAAUCAGCCAUAUCACAUCUGUAGAGGUUUUACUUGCUUUAAAAAACCUCCCACACCUCCCCCUGAACCUGAAACAUAAAAUGAAUGCAAUUGUUGUUGUUAACUUGUUUAUUGCAGCUUAUAAUGGUUACAAAUAAAGCAAUAGCAUCACAAAUUUCACAAAUAAAGCAUUUUUUUCACUGC

**Figure S1**
