# Supplementary figures and images for: Can ID Repetitive Elements Serve as Cis-acting Dendritic Targeting Elements? An In Vivo Study
Source: PLoS One. 2007 Sep 26;2(9):e961. doi: 10.1371/journal.pone.0000961 (PMC1978531; doi:10.1371/journal.pone.0000961)

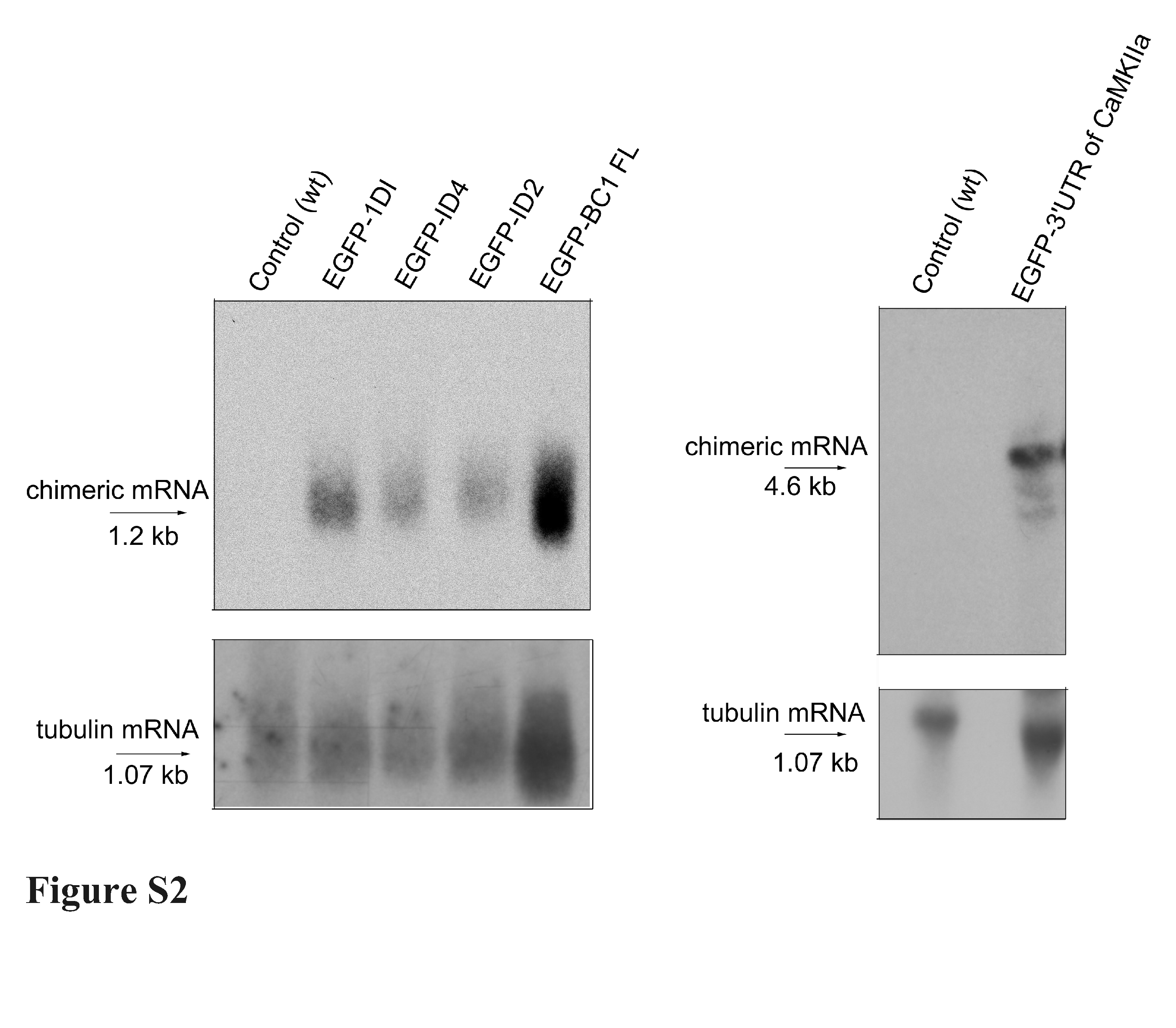

Supplement: Figure S2 — Chimeric mRNAs are Expressed in the Brain Northern blot hybridization of total RNA extracted from brain tissue of transgenic and wild type mice. The extracted RNAs were separated on a 1.2% denaturing agarose gel and hybridized with 32P-labeled probe complementary to EGFP. Specific signals corresponding to the expected sizes was observed for chimeric RNAs. As a loading control the membrane was hybridized with a probe complimentary to α-tubulin mRNA. (7.73 MB DOC) [file pone.0000961.s002.doc]

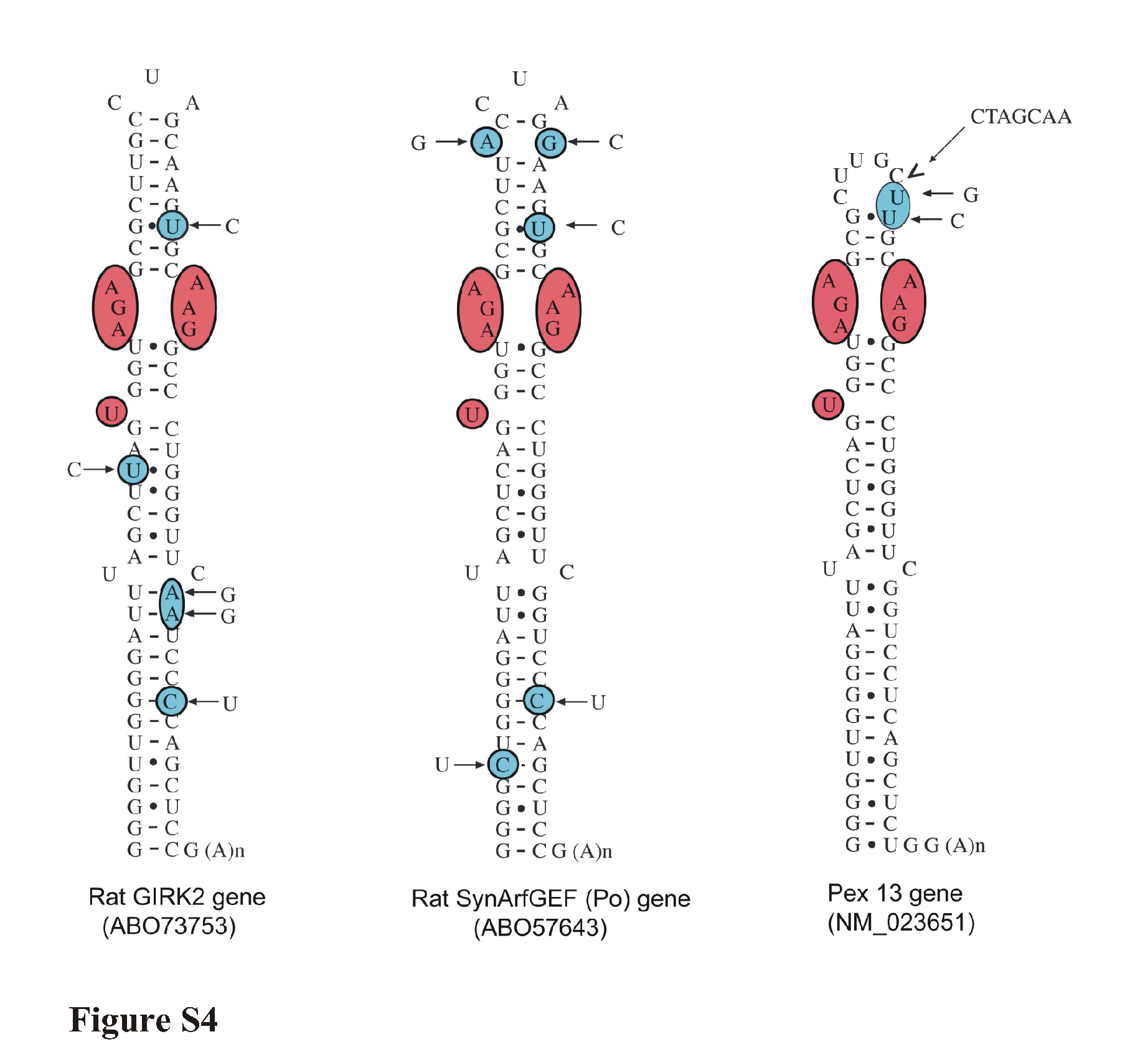

Supplement: Figure S4 — Secondary structures of ID elements. Secondary structures of the ID domains found in other genes and for which brain in situ hybridization data are available in the literature or in databases, respectively. Nucleotides corresponding to those thought to be vital for dendritic transport (see above) are highlighted in red and deviations from the ID element in BC1 RNA in blue. The corresponding nucleotides in BC1 RNA are indicated by arrows. (9.35 MB TIF) [file pone.0000961.s004.tif]
