# Supplementary material for: Can ID Repetitive Elements Serve as Cis-acting Dendritic Targeting Elements? An In Vivo Study
Source: PLoS One. 2007 Sep 26;2(9):e961. doi: 10.1371/journal.pone.0000961 (PMC1978531; doi:10.1371/journal.pone.0000961)
Supplement: Figure S3 — Sequences of ID elements from our chimeric RNAs and from published examples found in the UTRs of other genes. Alignment of ID elements to the one found in the 5′ domain of dendritic BC1 RNA in rat (Rno, top line) and mouse (Mmu, second line). Nucleotides identical to the ID domain of rat BC1 RNA are shown as dots, nucleotide replacements by the corresponding changes, and deletions by hyphens. The areas thought to be vital for dendritic transport [38] are in bold lettering. The ID1, ID2, and ID4 elements all fold in the same manner as does the corresponding BC1 RNA domain [47]. (0.02 MB DOC) [file pone.0000961.s003.doc]

10 20 30 40

. . . .

**BC1_Rno** 5’ GGGGUUGGGGAUUUAGCUCAG**U**GGU**AGA**GCGCUUGCCUAGC**AAG**

**BC1_Mmu** GGGGUUGGGGAUUUAGCUCAG**U**GGU**AGA**GCGCUUGCCUAGC**AAG**

**ID1_Rno** ............................................

**ID2_Rno** ............................................

**ID4_Rno** ....C.............................A.....G...

**GIRK2_Rno** ..................U.........................

**SynArfGEF_Rno** ....C.............................A.....G...

**Pex13_Mmu** ....................................-------U

50 60 70

. . .

**BC1_Rno** CGCAAGGCCCUGGGUUCGGUCCUCAGCUCCGAA

**BC1_Mmu** CGCAAGGCCCUGGGUUCGGUCCUCAGCUC*U*G*G*A

**ID1_Rno** .................................

**ID2_Rno** ......................C..........

**ID4_Rno** ......................C..........

**GIRK2_Rno** U................AA...C..........

**SynArfGEF_Rno** C.....................C..........

**Pex13_Mmu** U............................U.G.

**Figure S3**
